# Distinct clinical profiles and post-transplant outcomes among kidney transplant recipients with lower education levels: uncovering patterns through machine learning clustering

**DOI:** 10.1080/0886022X.2023.2292163

**Published:** 2023-12-12

**Authors:** Charat Thongprayoon, Jing Miao, Caroline Jadlowiec, Shennen A. Mao, Michael Mao, Napat Leeaphorn, Wisit Kaewput, Pattharawin Pattharanitima, Oscar A. Garcia Valencia, Supawit Tangpanithandee, Pajaree Krisanapan, Supawadee Suppadungsuk, Pitchaphon Nissaisorakarn, Matthew Cooper, Wisit Cheungpasitporn

**Affiliations:** a Division of Nephrology and Hypertension, Department of Medicine, Mayo Clinic, Rochester, MN, USA; b Division of Transplant Surgery, Mayo Clinic, Phoenix, AZ, US; c Division of Transplant Surgery, Mayo Clinic, Jacksonville, FL, USA; d Division of Nephrology and Hypertension, Department of Medicine, Mayo Clinic, Jacksonville, FL, USA; e Department of Military and Community Medicine, Phramongkutklao College of Medicine, Bangkok, Thailand; f Department of Internal Medicine, Thammasat University, Pathum Thani, Thailand; g Chakri Naruebodindra Medical Institute, Ramathibodi Hospital, Mahidol University, Samut Prakan, Thailand; h Department of Medicine, Division of Nephrology, Massachusetts General Hospital, Harvard Medical School, Boston, MA, USA; i Division of Transplant Surgery, Medical College of Wisconsin, Milwaukee, WI, USA

**Keywords:** Low education level, kidney transplant, post-transplantation outcome, clustering, machine learning

## Abstract

**Background:**

Educational attainment significantly influences post-transplant outcomes in kidney transplant patients. However, research on specific attributes of lower-educated subgroups remains underexplored. This study utilized unsupervised machine learning to segment kidney transplant recipients based on education, further analyzing the relationship between these segments and post-transplant results.

**Methods:**

Using the OPTN/UNOS 2017–2019 data, consensus clustering was applied to 20,474 kidney transplant recipients, all below a college/university educational threshold. The analysis concentrated on recipient, donor, and transplant features, aiming to discern pivotal attributes for each cluster and compare post-transplant results.

**Results:**

Four distinct clusters emerged. Cluster 1 comprised younger, non-diabetic, first-time recipients from non-hypertensive younger donors. Cluster 2 predominantly included white patients receiving their first-time kidney transplant either preemptively or within three years, mainly from living donors. Cluster 3 included younger re-transplant recipients, marked by elevated PRA, fewer HLA mismatches. In contrast, Cluster 4 captured older, diabetic patients transplanted after prolonged dialysis duration, primarily from lower-grade donors. Interestingly, Cluster 2 showcased the most favorable post-transplant outcomes. Conversely, Clusters 1, 3, and 4 revealed heightened risks for graft failure and mortality in comparison.

**Conclusions:**

Through unsupervised machine learning, this study proficiently categorized kidney recipients with lesser education into four distinct clusters. Notably, the standout performance of Cluster 2 provides invaluable insights, underscoring the necessity for adept risk assessment and tailored transplant strategies, potentially elevating care standards for this patient cohort.

## Introduction

The transplantation of organs plays a crucial role in treating individuals suffering from end-stage organ failure. Nevertheless, patients with lower education levels or lower socioeconomic status may encounter obstacles that can adversely affect their post-transplantation outcomes [1–3]. Research findings indicate that individuals lacking private insurance or a stable source of income may encounter challenges in accessing healthcare services, including the opportunity for transplantation [1,2,4].

Education level is a potential barrier in kidney transplantation [5], while a study suggested that limited health literacy was strongly associated with reduced access to kidney transplantation [6]. Individuals with greater socioeconomic status and higher levels of education are found to have a higher likelihood of choosing living donors for transplan­tation [7,8]. Moreover, several studies have indicated that education level could serve as a significant predictor of post-transplant outcomes among recipients [9]. Lower education levels have been associated with increased risks of graft failure [10–12], delayed graft function [12], patient mortality [1,10–12], and non-adherence to medication regimens [13,14]. This group of recipients may also possess distinct clinical characteristics compared to individuals with higher education levels or socioeconomic status, which can contribute to inferior outcomes. However, there is a scarcity of research investigating the diversity among transplant recipients based on their education level.

The utilization of artificial intelligence and machine learning (ML) has proved valuable in organ transplantation research, allowing for the identification of unique subtypes and novel data patterns [15–18]. Among ML techniques, unsupervised consensus clustering stands out as an effective approach that can unveil similarities and heterogeneities within diverse data variables. By categorizing these variables into clinically relevant clusters, this method offers new perspectives and insights for clinical nephrologists [19–22]. Recent studies have shown that distinct subtypes identified by the ML consensus clustering approach can predict distinct clinical outcomes [23–27]. Therefore, employing ML consensus clustering tool becomes increasingly promising in identifying specific phenotypes of transplant recipients based on education level. Gaining a comprehensive understanding of these diverse phenotypes among transplant recipients with lower education levels can empower the transplant community to develop targeted strategies aimed at improving outcomes within this patient population.

This study employed an unsupervised ML clustering technique to examine the UNOS/OPTN database spanning the years 2017 to 2019. The primary objective was to identify distinct clusters of kidney transplant recipients characterized by lower education levels and subsequently evaluate the clinical outcomes within each cluster.

## Materials and methods

This study was approved by the Mayo Clinic Institutional Review Board (21-007698). To conduct this study, we utilized the Organ Procurement and Transplantation Network (OPTN)/United Network for Organ Sharing (UNOS) database, which contains information on kidney transplant recipients aged 18 years or older in the United States between 2017 and 2019. Specifically, we included recipients who indicated their highest education level as ‘none,’ ‘grade school (0–8),’ or ‘high school (9-10) or equivalent test.’

To perform clustering analysis, we extracted relevant recipient-, donor-, and transplant-related characteristics from the database. All variables demonstrated missing data below 5%, which we addressed using the Multiple Imputation by Chained Equations (MICE) method, employing 10 imputed datasets to ensure statistical robustness and computational practicality. This number of imputations was selected to effectively represent the uncertainty due to missing data while maintaining computational efficiency (**Supplementary Table 1**).

### Clustering analysis

We employed an unsupervised ML technique, specifically a consensus clustering approach [28], to categorize clinical phenotypes of kidney transplant recipients with lower education degrees. We input recipient-, donor-, and transplant-related characteristics into clustering analysis as either continuous or categorical variables as shown in Table 1. To ensure meaningful clinical outcomes, we set a subsampling parameter of 80%, drawing on the principles of bootstrapping to introduce randomness and enhance generalizability. We performed 100 iterations to strike a balance between computational feasibility and the robustness of the clustering outcomes. Additionally, we explored the number of potential clusters (k) ranging from 2 to 10, a range informed by the expected heterogeneity in our dataset and commonly used in clinical data analyses to identify patient subgroups without overcomplication. We evaluated the optimal number of clusters by examining the consensus matrix (CM) heat map, cumulative distribution function (CDF), cluster-consensus plots, within-cluster consensus scores, and the proportion of ambiguously clustered pairs (PAC). The within-cluster consensus score, ranging from 0 to 1, indicates cluster stability, with values closer to 1 representing higher stability [19]. PAC, ranging from 0 to 1, measures cluster stability as the proportion of sample pairs with consensus values falling within the predetermined boundaries, with values closer to 0 indicating better stability [29]. Detailed information on the consensus cluster algorithms employed in this study can be found in the Online Supplementary.

**Table 1.** Clinical characteristics according to clusters of kidney transplant recipients with education under college/university level.

	All	Cluster 1	Cluster 2	Cluster 3	Cluster 4	p-value
	(*n* = 20474)	(*n* = 6239)	(*n* = 4493)	(*n* = 1866)	(*n* = 7876)	
Recipient Age (year)	51.6 ± 13.9	43.6 ± 12.5	50.4 ± 14.5	45.7 ± 14.3	59.9 ± 8.8	<0.001
Recipient male sex	12722 (62.1)	3601 (57.7)	2923 (65.1)	1108 (59.4)	5090 (64.6)	<0.001
Recipient race						
- White - Black - Hispanic - Other	7445 (36.4)	1696 (27.2)	2597 (57.8)	828 (44.4)	2324 (29.5)	<0.001
	5685 (27.8)	2095 (33.6)	513 (11.4)	468 (25.1)	2609 (33.1)	<0.001
	5712 (27.9)	1858 (29.8)	1148 (25.6)	453 (24.3)	2253 (28.6)	<0.001
	1632 (8.0)	590 (9.5)	235 (5.2)	117 (6.3)	690 (8.8)	<0.001
Body mass index (kg/m^2^)	28.5 ± 5.5	27.4 ± 5.6	28.5 ± 5.4	26.7 ± 5.4	29.8 ± 5.2	<0.001
Kidney retransplant	1929 (9.4)	0 (0.0)	49 (1.1)	1864 (99.9)	16 (0.2)	<0.001
Dialysis duration						
- Preemptive - <1 year - 1-3 years - >3 years	2582 (12.6)	542 (8.7)	1354 (30.1)	211 (11.3)	475 (6.0)	<0.001
	2161 (10.6)	420 (6.7)	1038 (23.1)	214 (11.5)	489 (6.2)	
	4473 (21.8)	1219 (19.5)	1377 (30.6)	469 (25.1)	1408 (17.9)	
	11258 (55.0)	4058 (65.0)	724 (16.1)	972 (52.1)	5504 (69.9)	
Cause of end-stage kidney disease						
- Diabetes mellitus - Hypertension - Glomerular disease - PKD - Other	6006 (29.3)	421 (6.7)	1208 (26.9)	66 (3.5)	4311 (54.7)	<0.001
	5076 (24.8)	2276 (36.5)	869 (19.3)	165 (8.8)	1766 (22.4)	<0.001
	4065 (19.9)	1884 (30.2)	1178 (26.2)	270 (14.5)	733 (9.3)	<0.001
	1453 (7.1)	483 (7.7)	505 (11.2)	42 (2.3)	423 (5.4)	<0.001
	3874 (18.9)	1175 (18.8)	733 (16.3)	1323 (70.9)	643 (8.2)	<0.001
Comorbidity						
- Diabetes mellitus - Malignancy - Peripheral vascular disease	7408 (36.2)	629 (10.1)	1471 (32.7)	336 (18.0)	4972 (63.1)	<0.001
	1536 (7.5)	301 (4.8)	343 (7.6)	182 (9.8)	710 (9.0)	<0.001
	2021 (9.8)	282 (4.5)	424 (9.4)	128 (6.9)	1178 (15.0)	<0.001
PRA, median (IQR)	0 (0-37)	0 (0-42)	0 (0-1)	93 (50-100)	0 (0-21)	<0.001
Positive HCV serostatus	1042 (5.1)	272 (4.4)	95 (2.1)	88 (4.7)	587 (7.5)	<0.001
Positive HBs antigen	268 (1.3)	98 (1.6)	41 (0.9)	30 (1.6)	99 (1.3)	0.02
Positive HIV serostatus	238 (1.2)	109 (1.7)	28 (0.6)	5 (0.3)	96 (1.2)	<0.001
Functional status						<0.001
- 10-30% - 40-70% - 80-100%	37 (0.2)	9 (0.1)	6 (0.1)	3 (0.2)	19 (0.2)	
	10351 (50.6)	3078 (49.3)	1736 (38.6)	962 (51.6)	4575 (58.1)	
	10086 (49.3)	3152 (50.5)	2751 (61.2)	901 (48.3)	3282 (41.7)	
Working income	5040 (24.6)	1696 (27.2)	1853 (41.2)	472 (25.3)	1019 (12.9)	<0.001
Public insurance	15709 (76.7)	4943 (79.2)	2349 (52.3)	1486 (79.6)	6931 (88.0)	<0.001
US resident	19979 (97.6)	5952 (95.4)	4395 (97.8)	1843 (98.8)	7789 (98.9)	<0.001
Education						<0.001
- None - Grade school - High school	218 (1.1)	55 (0.9)	25 (0.6)	12 (0.6)	126 (1.6)	
	2681 (13.1)	751 (12.0)	399 (8.9)	154 (8.3)	1377 (17.5)	
	17575 (85.8)	5433 (87.1)	4069 (90.6)	1700 (91.1)	6373 (80.9)	
Serum albumin (g/dL)	4.0 ± 0.5	4.1 ± 0.6	3.9 ± 0.5	3.9 ± 0.6	4.0 ± 0.5	<0.001
Kidney donor status						<0.001
- Non-ECD deceased - ECD deceased - Living	13622 (66.5)	6157 (98.7)	162 (3.6)	1475 (79.0)	5828 (74.0)	
	2171 (10.6)	32 (0.5)	36 (0.8)	102 (5.5)	2001 (25.4)	
	4681 (22.9)	50 (0.8)	4295 (95.6)	289 (15.5)	47 (0.6)	
ABO incompatibility	22 (0.1)	0 (0.0)	21 (0.5)	1 (0.1)	0 (0.0)	<0.001
Donor age (years)	39.5 ± 14.8	27.7 ± 12.3	43.0 ± 12.5	36.8 ± 13.7	47.5 ± 11.6	<0.001
Donor male sex	11235 (54.9)	4193 (67.2)	1546 (34.4)	1041 (55.8)	4455 (56.6)	<0.001
Donor race						
- White - Black - Hispanic - Other	13508 (66.0)	3941 (63.2)	2885 (64.2)	1227 (65.8)	5455 (69.3)	<0.001
	2559 (12.5)	900 (14.4)	386 (8.6)	251 (13.5)	1022 (13.0)	<0.001
	3460 (16.9)	1102 (17.7)	1009 (22.5)	305 (16.3)	1044 (13.3)	<0.001
	947 (4.6)	296 (4.7)	213 (4.7)	83 (4.4)	355 (4.5)	0.87
History of hypertension in donor	4680 (22.9)	544 (8.7)	225 (5.0)	359 (19.2)	3552 (45.1)	<0.001
KDPI						
- Living donor - KDPI < 85 - KDPI ≥ 85	4681 (22.9)	50 (0.8)	4295 (95.6)	289 (15.5)	47 (0.6)	<0.001
	14476 (70.7)	6154 (98.6)	188 (4.2)	1539 (82.5)	6595 (83.7)	<0.001
	1317 (6.4)	35 (0.6)	10 (0.2)	38 (2.0)	1234 (15.7)	<0.001
Total HLA mismatch, median (IQR)	4 (3–5)	4 (4–5)	3 (2–5)	4 (2–5)	5 (4–5)	<0.001
Cold ischemia time (hours)	14.3 ± 10.1	15.8 ± 7.8	2.4 ± 3.2	15.8 ± 9.3	19.6 ± 8.9	<0.001
Kidney on pump	7780 (38.0)	2387 (38.3)	32 (0.7)	632 (33.9)	4729 (60.0)	<0.001
Delay graft function	4982 (24.3)	1258 (20.2)	146 (3.2)	481 (25.8)	3097 (39.3)	<0.001
Allocation type						
- Local - Regional - National	15695 (76.7)	4910 (78.7)	4467 (99.4)	1056 (56.6)	5262 (66.8)	<0.001
	2306 (11.3)	599 (9.6)	8 (0.2)	269 (14.4)	1430 (18.2)	<0.001
	2473 (12.1)	730 (11.7)	18 (0.4)	541 (29.0)	1184 (15.0)	<0.001
EBV status						
- Low risk - Moderate risk - High risk	136 (0.7)	64 (1.0)	45 (1.0)	14 (0.8)	13 (0.2)	<0.001
	18936 (92.5)	5696 (91.3)	4090 (91.0)	1710 (91.6)	7440 (94.5)	<0.001
	1402 (6.8)	479 (7.7)	358 (8.0)	142 (7.6)	423 (5.4)	<0.001
CMV status						<0.001
- D-/R- - D-/R+ - D+/R+ - D+/R-	2787 (13.6)	807 (12.9)	990 (22.0)	240 (12.9)	750 (9.5)	
	5516 (26.9)	1851 (29.7)	961 (21.4)	545 (29.2)	2159 (27.4)	
	3242 (15.8)	1072 (17.2)	757 (16.8)	277 (14.8)	1136 (14.4)	
	8929 (43.6)	2509 (40.2)	1785 (39.7)	804 (43.1)	3831 (48.6)	
Induction immunosuppression						
- Thymoglobulin - Alemtuzumab - Basiliximab - Other - No induction	12438 (60.8)	4048 (64.9)	2055 (45.7)	1357 (72.7)	4978 (63.2)	<0.001
	3359 (16.4)	1040 (16.7)	956 (21.3)	285 (15.3)	1078 (13.7)	<0.001
	3815 (18.6)	847 (13.6)	1185 (26.4)	147 (7.9)	1636 (20.8)	<0.001
	152 (0.7)	45 (0.7)	30 (0.7)	15 (0.8)	62 (0.8)	0.88
	1432 (7.0)	429 (6.9)	370 (8.2)	112 (6.0)	521 (6.6)	0.002
Maintenance Immunosuppression						
- Tacrolimus - Cyclosporine - Mycophenolate - Azathioprine - mTOR inhibitors - Steroid	18708 (91.4)	5715 (91.6)	4156 (92.5)	1699 (91.1)	7138 (90.6)	0.004
	153 (0.7)	38 (0.6)	50 (1.1)	12 (0.6)	53 (0.7)	0.01
	18494 (90.3)	5671 (90.9)	4089 (91.0)	1651 (88.5)	7083 (89.9)	0.004
	66 (0.3)	11 (0.2)	26 (0.6)	10 (0.5)	19 (0.2)	0.001
	65 (0.3)	18 (0.3)	13 (0.3)	9 (0.5)	25 (0.3)	0.60
	13993 (68.3)	4400 (70.5)	2735 (60.9)	1469 (78.7)	5389 (68.4)	<0.001

Abbreviations: BMI: body mass index; CMV: cytomegalovirus; D: donor; EBV: Epstein-barr virus; ECD: extended criteria donor; HBs: hepatitis B surface; HCV: hepatitis C virus; HIV: human immunodeficiency virus; KDPI: kidney donor profile index; mTOR: mammalian target of rapamycin; PKD: polycystic kidney disease; PRA: panel reactive antibody; R: recipient.

### Outcomes

The posttransplant outcomes examined in this study included death-censored graft failure and patient mortality within 2 years after kidney transplant. Death-censored graft failure was defined as the need for dialysis or kidney re-transplantation, with censoring for patient death or the last reported follow-up date in the OPTN/UNOS database. We also analyzed acute allograft rejection within 1 year after kidney transplant. The OPTN/UNOS database indicated whether allograft rejection occurred within the specified timeframe but did not provide the exact occurrence date.

### Statistical analysis

After performing consensus clustering analysis to assign clusters to kidney transplant recipients with lower education levels, we compared clinical characteristics and posttransplant outcomes among the assigned clusters. We used the Chi-squared test for categorical characteristics and analysis of variance (ANOVA) for continuous characteristics to test differences in clinical characteristics among the assigned clusters. To identify distinct characteristics of each assigned cluster, we utilized the standardized mean difference with a pre-specified cutoff of >0.3. Patient survival and death-censored graft survival were estimated using Kaplan-Meier analysis, and comparisons among assigned clusters were conducted using the log-rank test. We calculated hazard ratios (HR) for patient death and death-censored graft failure using Cox proportional hazard analysis. Logistic regression analysis was employed to calculate odds ratios (OR) for acute allograft rejection. We adjusted HR and OR for recipient age, sex, race, body mass index, retranstplant status, caused of end-stage kidney disease, comorbidities, panel reactive antibody, hepatitis B, hepatitis C, HIV serostatus, functional status, serum albumin, decreased donor type, donor age, sex, race, hypertension, kidney donor profile index, HLA mismatch, allocation type, EBV and CMV status, induction and maintenance immunosuppression. All analyses were performed using R, version 4.0.3 (RStudio, Inc., Boston, MA; http://www.rstudio.com/), utilizing the ConsensusClusterPlus package (version 1.46.0) for consensus clustering analysis and the MICE command in R for multivariable imputation by chained equation.

## Result

### Identification of distinct clusters

There were 47,939 adult kidney transplant recipients in United States from 2017 to 2019. Of these, 20,474 (43%) reported their highest education levels under college or university at the time of kidney transplant. Therefore, we performed consensus clustering analysis in a total of 20,474 kidney transplant recipients with lower education levels. Table 1 showed recipient-, donor-, and transplant-related characteristics of included patients. The mean age was 52 ± 14 years and 62% were male. The majority of recipients were nonwhite (28% black, 28% Hispanic, and 8% other). Most patients had attained a high school education level (86%), while 13% had a grade school level and 1% had no education.

**Figure 1.**
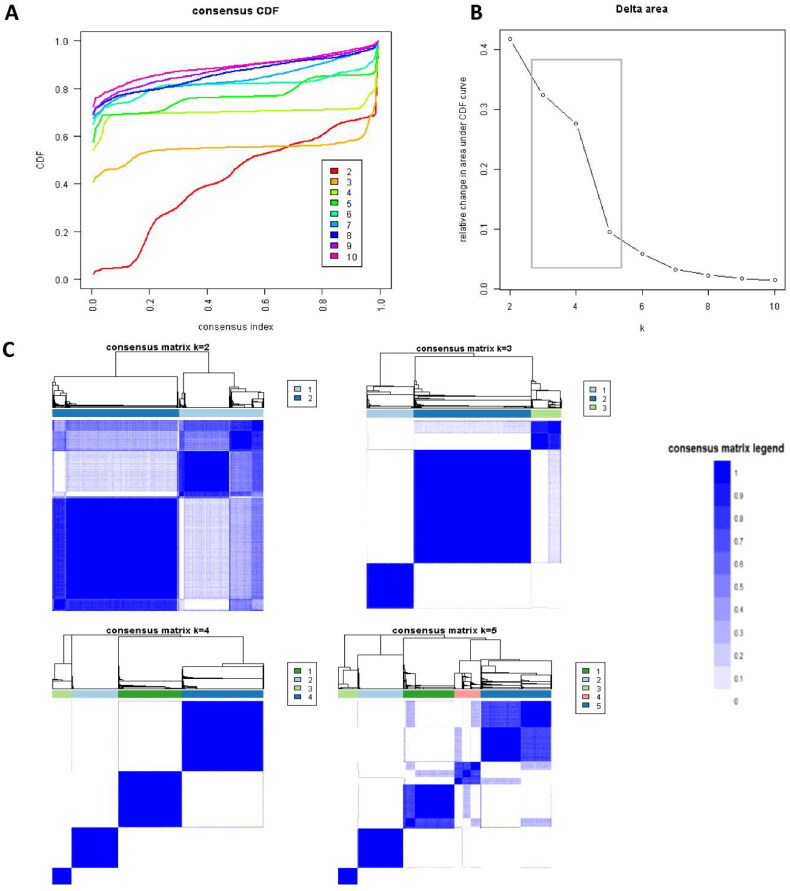
**A**. CDF plot displaying consensus distributions for each k; **B.** Delta area plot reflecting the relative changes in the area under the CDF curve. **C.** Consensus matrix heat map depicting consensus values on a white to blue color scale of each cluster.

**Figure 2.**
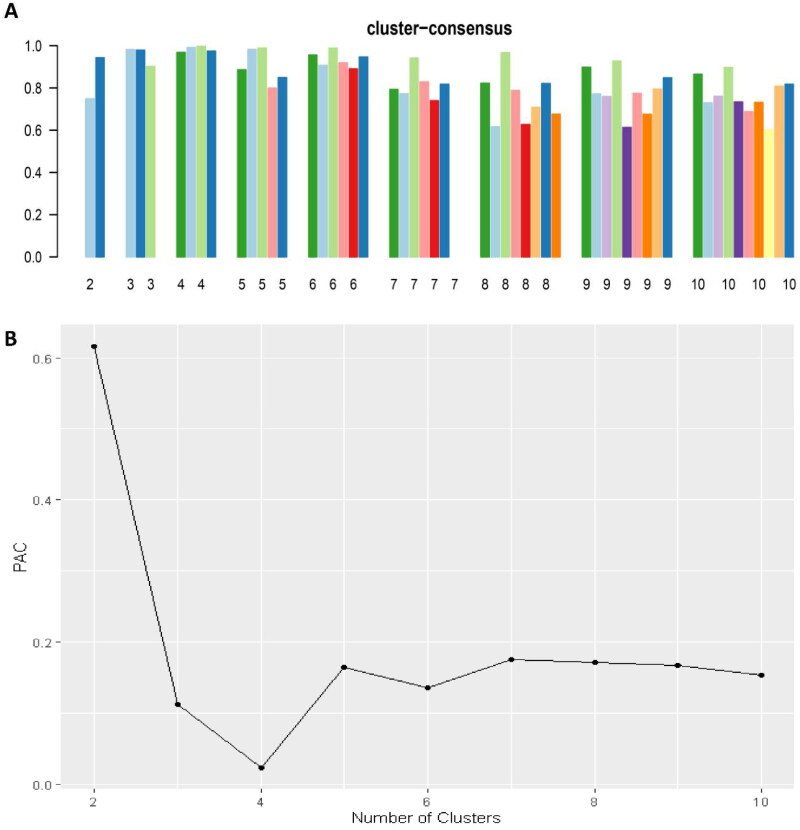
**A.** The bar plot represents the mean consensus score for different numbers of clusters (K ranges from two to ten); **B**. The PAC values assess ambiguously clustered pairs.

Figure 1A illustrates the CDF plot, which displays the consensus distributions for each cluster of kidney transplant recipients who had attained a lower education level. The delta area plot, depicted in Figure 1B, demonstrates the relative change in the area under the CDF curve. Notably, the most substantial changes in area occurred between *k* = 3 and *k* = 5, after which the relative increase in area became less pronounced. The CM heat map (Figure 1C, **Supplementary Figures 1-9**) revealed clear boundaries for cluster 3 and cluster 4, indicating robust cluster stability across multiple iterations. By analyzing the mean cluster consensus score (Figure 2A), it was found that cluster 4 exhibited the highest score. Furthermore, the evaluation of PACs using both strict and relaxed criteria displayed favorable low values for four clusters (Figure 2B). Consequently, by leveraging baseline variables at the time of transplant, the consensus clustering analysis successfully identified four clusters that best represented the data pattern observed in our cohort of kidney transplant recipients.

### Characteristics of each cluster

There were 6239 (30%) patients in cluster 1, 4493 (22%) patients in cluster 2, 1866 (9%) patients in cluster 3, 7876 (38%) patients in cluster 4. These four clusters were significantly distinct, as demonstrated in Table 1. According to standardized mean differences, shown in Figure 3, cluster 1 was characterized by young (mean recipient age 44 years), non-diabetic (90%) patients who received first-time (100%) kidney transplant from young (mean donor age 28 years), non-hypertensive (93%), and non-Expanded Criteria Donor (ECD) deceased donors (99%). The majority of cluster 1 recipients had >3 years of qualifying time (65%). Nearly all recipients (99%) received standard kidney donor profile index (KDPI) allograft (KDPI < 85%). Cluster 2 consisted of predominantly white patients (57%) who received first-time (99%) kidney transplant preemptively (30%) or with <3 years of dialysis (54%). Only 16% of cluster 2 recipients had >3 years of time on dialysis. Compared to other clusters, this cluster had more working employment (41%), less public insurance (52%), lower panel reactive antibody (PRA), a lower number of total HLA mismatches, shorter cold ischemia time, reduced use of kidney-perfused machine, and a lower incidence of delayed graft function. Nearly all patients (99.4%) in cluster 2 received kidney from local allocation. Cluster 3 was primarily characterized by young (mean recipient age 46 years) kidney re-transplant (99.9%) recipients, who exhibited a higher PRA and a lower number of total HLA mismatches. Cluster.3 recipients were more likely to receive kidney from national allocation in comparison with other clusters (29%). By comparison, patients in Cluster 4 were older (mean recipient age 60 years), more likely to be diabetic (63%), and have more than 3 years of dialysis (70%) time. Most recipients in cluster 4 received non-ECD (74%) standard KDPI (83.7%) kidneys although cluster 4 had the highest percentage of ECD (25%) and high KDPI (KDPI > 85%, 16%) utilization among the 4 clusters. Donors in cluster 4 exhibited certain characteristics distinguishing from donors in other clusters. They had a higher mean age of 48 years, a higher prevalence of hypertension (45%), a large proportion of ECD deceased donor (25%), and a higher percentage (16%) classified as high-KDPI with KDPI ≥ 85%.

Figure 3.**A.** The standardized differences across four clusters for each of baseline parameters. The x axis is the standardized differences value, and the y axis shows baseline parameters. The dashed vertical lines represent the standardized differences cutoffs of < −0.3 or > 0.3. Abbreviations: BMI: body mass index; CMV: cytomegalovirus; D: donor, DGF: delayed graft function, DM: diabetes mellitus, EBV: Epstein-Barr virus; ECD: extended criteria donor; ESKD:end-stage kidney disease; GN: glomeruloneph;itis, HBs: hepatitis B surface; HCV: hepatitis C virus; HIV: human immunodeficiency virus; HLA: human leucocyte antigen; HTN: hypertension; KDPI: kidney donor profile index; mTOR: mammalian target of rapamycin; PKD: polycystic kidney disease; PRA: panel reactive antibody; PVD: peripheral vascular disease; R: recipient. **B.** The standardized differences across four clusters for each of baseline parameters. The x axis is the standardized differences value, and the y axis shows baseline parameters. The dashed vertical lines represent the standardized differences cutoffs of < −0.3 or > 0.3. Abbreviations: BMI: Body mass index, CMV: cytomegalovirus, D: donor, DGF: delayed graft function, DM: diabetes mellitus, EBV: Epstein-Barr virus, ECD: extended criteria donor, ESKD: end-stage kidney disease, GN: glomerulonephritis, HBs: hepatitis B surface, HCV: hepatitis C virus; HIV: human immunodeficiency virus; HLA: human leucocyte antigen; HTN: hypertension; KDPI: kidney donor profile index; mTOR: mammalian target of rapamycin; PKD: polycystic kidney disease; PRA: panel reactive antibody; PVD: peripheral vascular disease; R: recipient.

**Figure:**
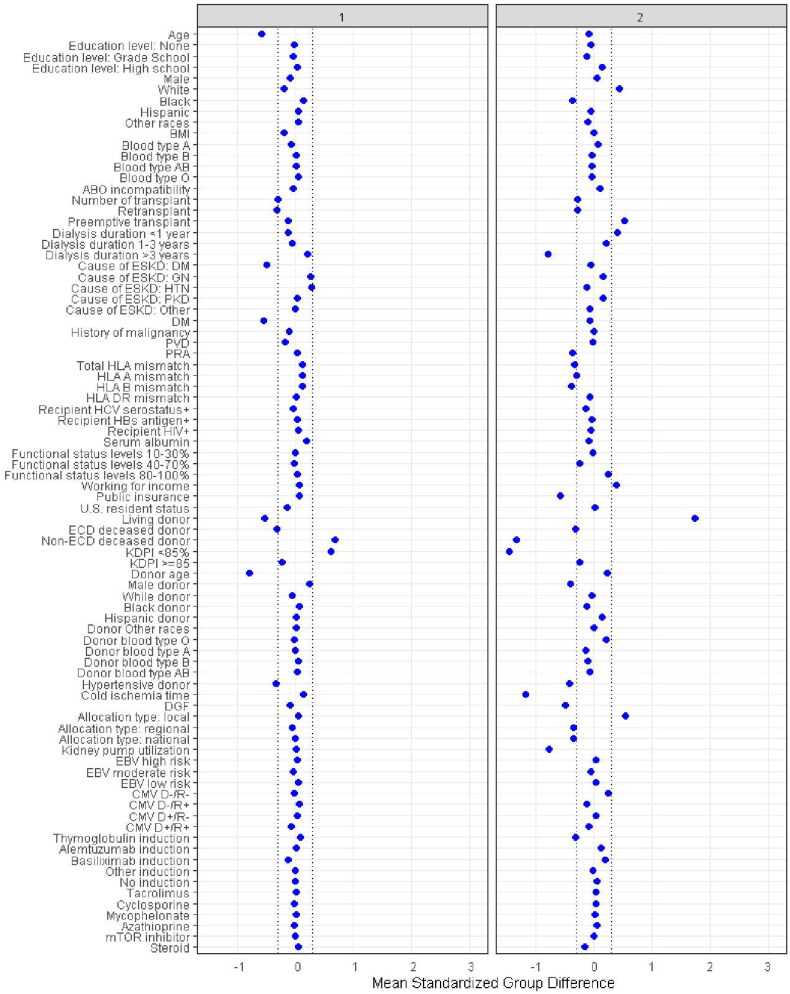

**Figure:**
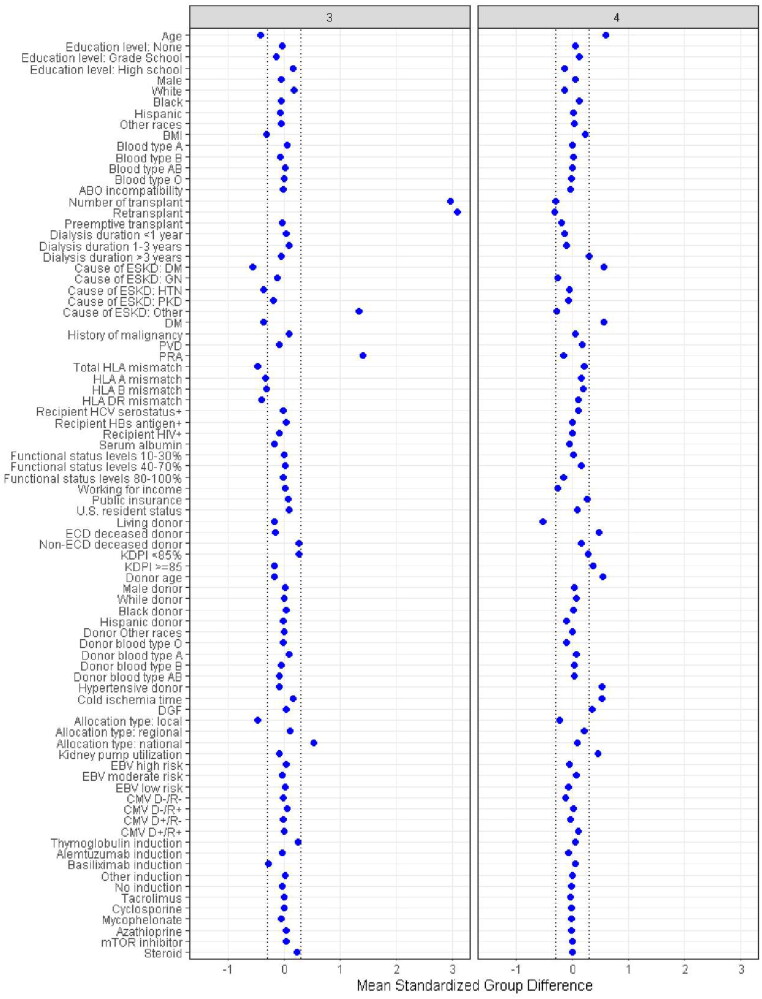

Supplementary Table 2 showed the distribution of clusters by UNOS regions. Region 6 and region 9 had the highest and lowest proportion of cluster 1 respectively. Region 7 and region 6 had the highest and lowest proportion of cluster 2 respectively. The proportion of cluster 3 was similar across regions. Region 11 and region 7 had the highest and lowest proportion of cluster 4 respectively (**Supplementary Table 2**).

### Posttransplant outcomes of each cluster

**Figure 4.**
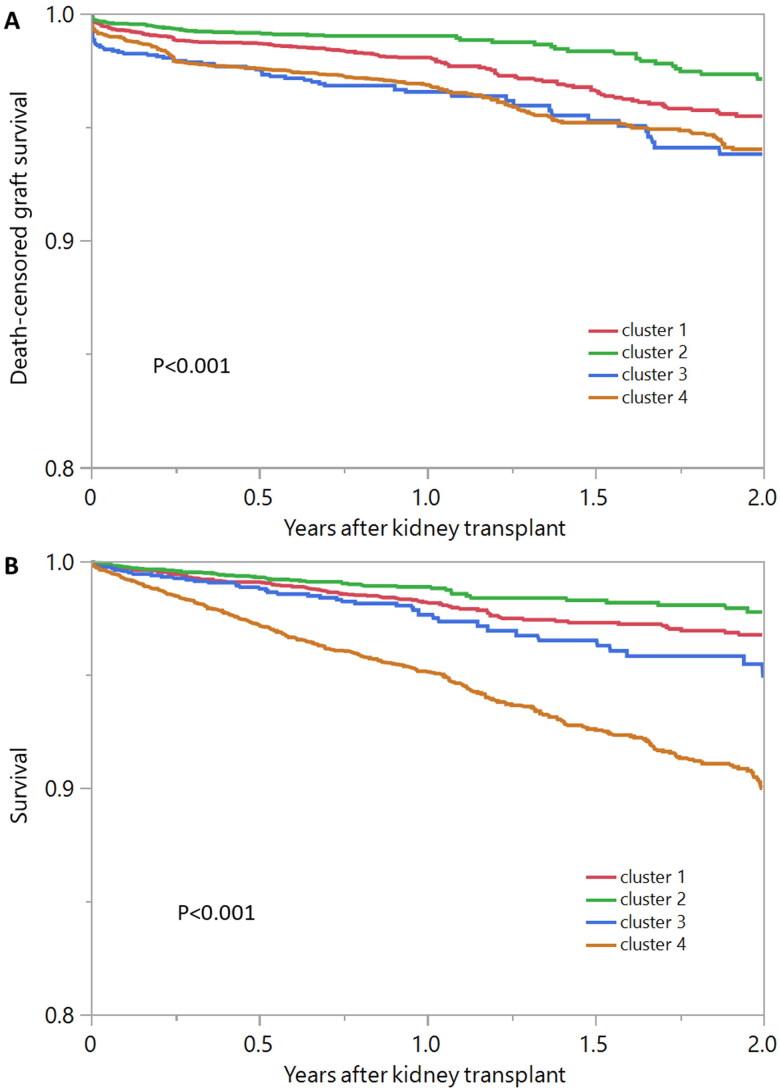
A) Death-censored graft survival and B) patient survival after kidney transplant among four unique clusters of kidney transplant recipients with lower degree of education in the U.S.

The risk of 2-year death-censored graft failure was observed to be 4.5% in cluster 1, 2.9% in cluster 2, 6.2% in cluster 3, and 6.0% in cluster 4 (*p* < 0.001) (Figure 4A). Additionally, the 2-year patient mortality risk were 3.3% in cluster 1, 2.3% in cluster 2, 5.2% in cluster 3, and 10% in cluster 4 (*p* < 0.001) (Figure 4B). Pairwise p-values showed significant difference in death-censored graft failure and patient death between assigned clusters except death-censored graft between cluster 3 and cluster 4, and death between cluster 1 and 3 (**Supplementary Table 3**). Cluster 1, cluster 3, and cluster 4 exhibited a significantly higher risk of death-censored graft failure and patient mortality when compared to cluster 2 in unadjusted and adjusted analyses (Table 2).

**Table 2.** Post-transplant outcomes according to clusters of kidney transplant recipients with education under college/university level.

	Cluster 1	Cluster 2	Cluster 3	Cluster 4
Death-censored graft failure at 2 years	4.5%	2.9%	6.2%	6.0%
Unadjusted HR for death-censored graft failure	1.75 (1.29–2.39)	1 (ref)	2.83 (1.98–4.03)	2.62 (1.96–3.50)
*Adjusted HR for death-censored graft failure	1.57 (1.08–2.28)	1 (ref)	2.43 (1.59–3.73)	1.58 (1.09–2.29)
Death at 2 years	3.3%	2.3%	5.2%	10.0%
Unadjusted HR for death	1.48 (1.06–2.06)	1 (ref)	2.02 (1.35–3.03)	4.27 (3.19–5.72)
*Adjusted HR for death	2.48 (1.65–3.71)	1 (ref)	2.24 (1.41–3.57)	2.20 (1.56–3.11)
Acute rejection in 1 years	3.9%	3.5%	5.5%	3.2%
Unadjusted OR for acute rejection	1.11 (0.91–1.37)	1 (ref)	1.62 (1.26–2.10)	0.91 (0.74–1.12)
*Adjusted OR for acute rejection	1.03 (0.78–1.34)	1 (ref)	1.15 (0.83–1.59)	0.77 (0.58–1.02)

Abbreviations: HR: hazard ratio; OR: odds ratio.

Adjusted for recipient age, sex, race, body mass index, retranstplant status, caused of end-stage kidney disease, comorbidities, panel reactive antibody, hepatitis B, hepatitis C, HIV serostatus, functional status, serum albumin, decreased donor type, donor age, sex, race, hypertension, kidney donor profile index, HLA mismatch, allocation type, EBV and CMV status, induction and maintenance immunosuppression.

The incidence of 1-year acute allograft rejection was found to be 3.9% in cluster 1, 3.5% in cluster 2, 5.5% in cluster 3, and 3.2% in cluster 4. Cluster 3 exhibited a significantly higher risk of acute rejection rate compared to cluster 2 in unadjusted analysis. However, the higher risk was attenuated and no longer statistically significant after multivariable adjustment. The risk of acute rejection in cluster 1 and cluster 4 was comparable to cluster 2.

## Discussion

In this study, a total of 20,474 kidney transplant recipients with lower education levels were identified for clustering. In the whole cohort, the majority of patients were nonwhite and had attained a high school education level. By using unsupervised ML approach, four distinct groups were successfully clustered, each characterized by unique clinical profiles and varying posttransplant outcomes.

Cluster 2 presented the most favorable post-transplant outcomes. This cluster was characterized by a demographic where over half of the patients are white, yet a significant 42% are nonwhite, illustrating a diverse composition. Patients in this cluster typically received first-time kidney transplants either preemptively or within 3 years of initiating dialysis. Early referral to transplant prior to starting dialysis continues to disproportionately impact nonwhite patients as does access to living donor kidney transplantation. These disparities can be attributed to a combination of various socioeconomic and biological risk factors. Limiting time on dialysis, as well as having access to a living donor transplant, are factors strongly linked to favorable kidney transplant outcomes [30], Barriers to living donation for nonwhite patients have included increased ESRD risk within certain patient populations and ineligibility of potential donors [31–33]. Additionally, despite improved support services available to donors, including lost wage reimbursement, significant challenges remain for those donors needing to take time off work or those who are primary household caregivers, these challenges have been shown to disproportionately impact donors in lower income households [32,34]. In the most recent era, the median graft survival is around 19 years for living donor transplants and 12 years for deceased donor kidney transplants [35]. A recent study showed that the 5-year living donor graft loss was decreased by 66% compared to deceased donors [36]. Another study also showed a decreased risk of graft failure and a decreased risk of mortality among living donor graft recipients as compared to deceased graft recipients [37].

Cluster 3 and cluster 4 displayed the poorest post-transplant outcomes. In comparison to cluster 2, both clusters experienced a significant threefold increase in 2-year death-censored graft loss. Moreover, they exhibited higher risks of 2-year mortality, with cluster 1 being approximately twice as high and cluster 4 being four times as high compared to cluster 2. Cluster 3 stands out for several key characteristics. The causes for inferior patient and graft survival in clusters 3 and 4 likely differ. All recipients in this cluster were kidney re-transplant recipients with higher exposure to sensitizing events. Survival for those undergoing re-transplantation is often inferior is often attributed to immunologic and infectious risk. While there was a correlation between multiple kidney transplants and improved HLA matching at the time of transplantation (*p* < 0.0001), a difference in death-censored graft survival based on the number of transplants was observed. The median graft survival was 328 months for recipients of the first transplant, 209 months for the second transplant, and 150 months for the third transplant (*p* = 0.038) [38]. Secondly, they exhibited the highest PRA levels with a median 93%. Kidney transplant recipients with PRA of >50% had significantly higher risk of overall and death-censored graft failure and all-cause mortality independent of acute rejection, age and time on dialysis [39]. Compared to cluster 2, only cluster 3 showed a higher risk of 1-year acute rejection, a known risk factor for reduced death-censored graft survival [40]. Cluster 4 recipients were characterized by older, diabetic patients, with longer dialysis duration preceding transplantation. Limiting time on dialysis is beneficial and a pretransplant dialysis duration >24 months has been found to be a significant risk factor associated with poor death-censored graft survival [40]. Regardless of the origin of the donor organ, adjusted mortality rates are 75% higher with rates of adjusted graft losses 25% higher in diabetic recipients [41,42]. This may be more likely related to the highest 2-year mortality in this cluster. Compared to other 3 clusters, donor was older, and more had hypertension history (almost half) and high KDPI in cluster 4. It should be noted that donor hypertension increased the risk of post-transplant hypertension (OR 3.23; 95% CI 1.05–9.96) among kidney transplant recipients and also increased the risk of allograft failure (OR 1.31; 95% CI 1.06–1.63); however, donor hypertension was not a risk factor for mortality (OR 0.996; 95% CI 0.65–1.52) among renal transplant recipients [43]. In addition, the longest cold ischemia time and highest incidences of kidney on pump machine and delay graft function were found in cluster 4. Cold ischemia time was associated with a higher risk of delayed graft function, but this influence was relatively modest compared to the impact of the KDPI [44].

Cluster 1 displayed slightly elevated risks compared to cluster 2 in terms of 2-year death-censored graft loss (1.8-fold increase) and 2-year mortality (1.5-fold increase). Cluster 1 stood out with the lowest proportion of diabetic patients and the youngest recipient age among the four clusters. However, this cluster was notably characterized by non-ECD deceased donors, longer pretransplant dialysis duration exceeding 3 years, and higher incidences of kidney on pump machine usage and delayed graft function. These factors likely contribute to the observed inferior outcomes in cluster 1 [35,36,40,45].

The results of this study carry substantial implications for improving transplant outcomes in the future. By identifying specific patient and donor characteristics that can anticipate unfavorable results, clinicians can optimize treatment approaches and organ allocation. Future research endeavors should focus on identifying additional factors, including immunological and genetic components, that impact transplant outcomes. Additionally, interventions should be developed to enhance outcomes for high-risk patients. The study’s findings also propose various policy interventions that could enhance transplant outcomes for individuals with lower education levels in the United States. To improve transplant outcomes, targeted interventions should be implemented for different clusters. For cluster 1, which comprises young, non-diabetic patients receiving their first kidney transplant from specific deceased donors, and longer pretransplant dialysis duration exceeding 3 years, interventions should focus on enhancing pre-transplant education and counseling, ensuring timely access to transplantation, and optimizing donor selection criteria. For cluster 2, consisting of white patients receiving preemptive or early kidney transplants from living donors, policies should aim to overcome barriers to living donor transplants. This can be achieved by increasing awareness about of living donation benefits, providing financial incentives or support, and optimizing organ allocation strategies to minimize HLA mismatches and cold ischemia time. Addressing the suboptimal outcomes in cluster 3, which includes young recipients of kidney re-transplants, requires the development of specific strategies. These may involve prioritizing re-transplantation patients on the organ waiting list, ensuring comprehensive pre-transplant evaluation and counseling, and exploring innovative approaches to improve organ matching and reduce total HLA mismatch. Lastly, policies should be implemented to enhance outcomes for cluster 4, consisting of older, diabetic patients receiving kidney transplants from lower-quality donors. Measures such as improved donor screening and selection processes, comprehensive pre-transplant assessment, and targeted post-transplant care and monitoring should be adopted to address the unique needs of this patient group.

Ensuring equity and accessibility should be prioritized in kidney transplantation policies. This can be accomplished by promoting education and awareness within communities that have lower education levels, fostering collaboration among healthcare professionals, community organizations, and educational institutions, and guaranteeing equitable access to transplant evaluation, care, and support services for all patients. The study distinguished four clusters of kidney transplant recipients, each with unique characteristics and regional distributions. Cluster 1 included younger, non-diabetic, first-time recipients with organs from similarly young, non-hypertensive donors, most prevalent in Region 6. Cluster 2, primarily white patients receiving initial transplants from living donors, showed the best outcomes and was most common in Region 7. Cluster 3 comprised younger re-transplant recipients with higher PRA and fewer HLA mismatches, evenly distributed across regions. In contrast, Cluster 4 contained older, diabetic patients with transplants after extended dialysis, predominantly from lower-grade donors, with the highest incidence in Region 11 and the lowest in Region 7. These findings emphasize the importance of considering regional demographics and healthcare access in kidney transplantation policies. By implementing these policy recommendations, policymakers can actively strive to enhance transplant outcomes, diminish disparities, and secure equitable access to high-quality healthcare for kidney transplant recipients, taking into account the regional variations in patient characteristics and outcomes.

One limitation of this study is the absence of reported data on the reasons behind lower education levels from the UNOS. Several factors may contribute to the prevalence of lower education levels in the United States. Socioeconomic status plays a significant role, as individuals from lower-income households may face barriers to accessing quality educational resources, including schools, books, and tutoring services. Systemic racism and discrimination can also hinder education for marginalized communities, manifested through unequal funding for schools in low-income areas, limited diversity in curricula and faculty, and implicit bias in academic assessment and admissions processes. Family and cultural influences may also be influential, with some individuals coming from families that prioritize work over education to have limited cultural emphasis on academic achievement. Moreover, personal circumstances such as family responsibilities or health issues can make it challenging for individuals to prioritize or pursue higher education. Finally, academic difficulties such as learning disabilities or a lack of support in early childhood education may impede academic success and hinder the pursuit of higher education. The various reasons for lower education levels can impact the outcomes of studies, including the present research on kidney transplant recipients.

In our study, we utilized unsupervised ML techniques to group kidney transplant recipients with lower education levels into four distinct clusters based on various characteristics, each exhibiting different post-transplant outcomes. The findings of our study hold significant implications for personalized post-transplant care and monitoring. Particularly, the identification of cluster 2, with the most favorable outcomes, suggests the potential for risk stratification and tailored transplant management for this specific patient population. However, clusters 1, 3, and 4 exhibited higher rates of death-censored graft failure and patient mortality compared to cluster 2, underscoring the need for heightened attention to validate these findings and elucidate the underlying factors contributing to the observed disparities between clusters.

## Supplementary Material

Supplemental Material
